# Case report: Apalutamide-induced severe lethal cutaneous adverse effects in China

**DOI:** 10.3389/fimmu.2023.1291564

**Published:** 2024-01-11

**Authors:** Qi Wang, Huali Cao, Xuetong Zhang, Huifeng Wu, Zhuangli Tang

**Affiliations:** ^1^ Department of Dermatology, Second Affiliated Hospital of Zhejiang University, Hangzhou, China; ^2^ Department of Dermatology, Changxing People’s Hospital, Huzhou, China; ^3^ Department of Urology, Second Affiliated Hospital of Zhejiang University School of Medicine, Hangzhou, China

**Keywords:** apalutamide, severe dermatologic adverse events, long incubation period, prognosis, withdrawal

## Abstract

**Introduction:**

Apalutamide is a novel agent for castration-resistant prostate cancer while skin rashes are the most common untoward reactions. Up to now, most of the reported dermatologic adverse events (dAEs) allocated to mild and moderate with a fair prognosis. Herein, we report a case series of severe dAEs in China caused by apalutamide.

**Case presentation:**

The four patients all developed severe and lethal drug eruptions including Stevens-Johnson syndrome and toxic epidermal necrolysis with a mean incubation period of 40 days. On the basis of the medical condition, all the patients were suggested to withdraw apalutamide and three of them recovered. Of note, attempts of rechallenges of apalutamide may be fatal.

**Discussion:**

The incidence of dAEs in previously conducted clinical trials exceeded 20%, with maculopapular rashes being the most common feature. However, the incidence and severity varied in different geographic regions and ethnicities. Inadequate attention was paid to severe cutaneous adverse reactions. Long latency may easily lead to the misdiagnosis of dAEs, and immediate withdrawal of apalutamide is the cornerstone of therapies.

**Conclusion:**

Special and adequate attention should be paid to apalutamide-attributed severe cutaneous adverse effects. Besides, the prognosis of severe drug eruptions may be disappointing, and in-time withdrawal is vital.

## Introduction

Apalutamide, an androgen receptor antagonist, has recently been approved for the treatment of metastatic, castration-sensitive and non-metastatic, castration-resistant prostate cancer (PC) (1). On the basis of previous literature, dermatologic adverse events (dAEs) remain the most reported side effects of apalutamide. Up to now, 15 case reports and 3 retrospective analyses of apalutamide-related drug eruptions have been published, with a divergent geographic distribution indicating the Japanese most vulnerable to dAEs of apalutamide (2–15). However, most of the dAEs remain mild and moderate with a fair prognosis (16, 17). Globally, only five patients have been recorded as having experienced fatal outcomes due to a severe drug rash, i.e., Stevens-Johnson syndrome (SJS) and toxic epidermal syndrome (TEN), the most life-threatening conditions, which are relatively rare with the estimated incidence fluctuating around 0.94 to 5.76 per million persons per year (18–20), induced by apalutamide—four in Japan and one solitary case in China. Reports of severe dAEs or fatalities associated with apalutamide in China remained extremely rare. In this report, we documented four cases of severe dAEs including SJS and TEN and depicted the overall prognosis.

## Case description

### Case 1

**Figure 1:**
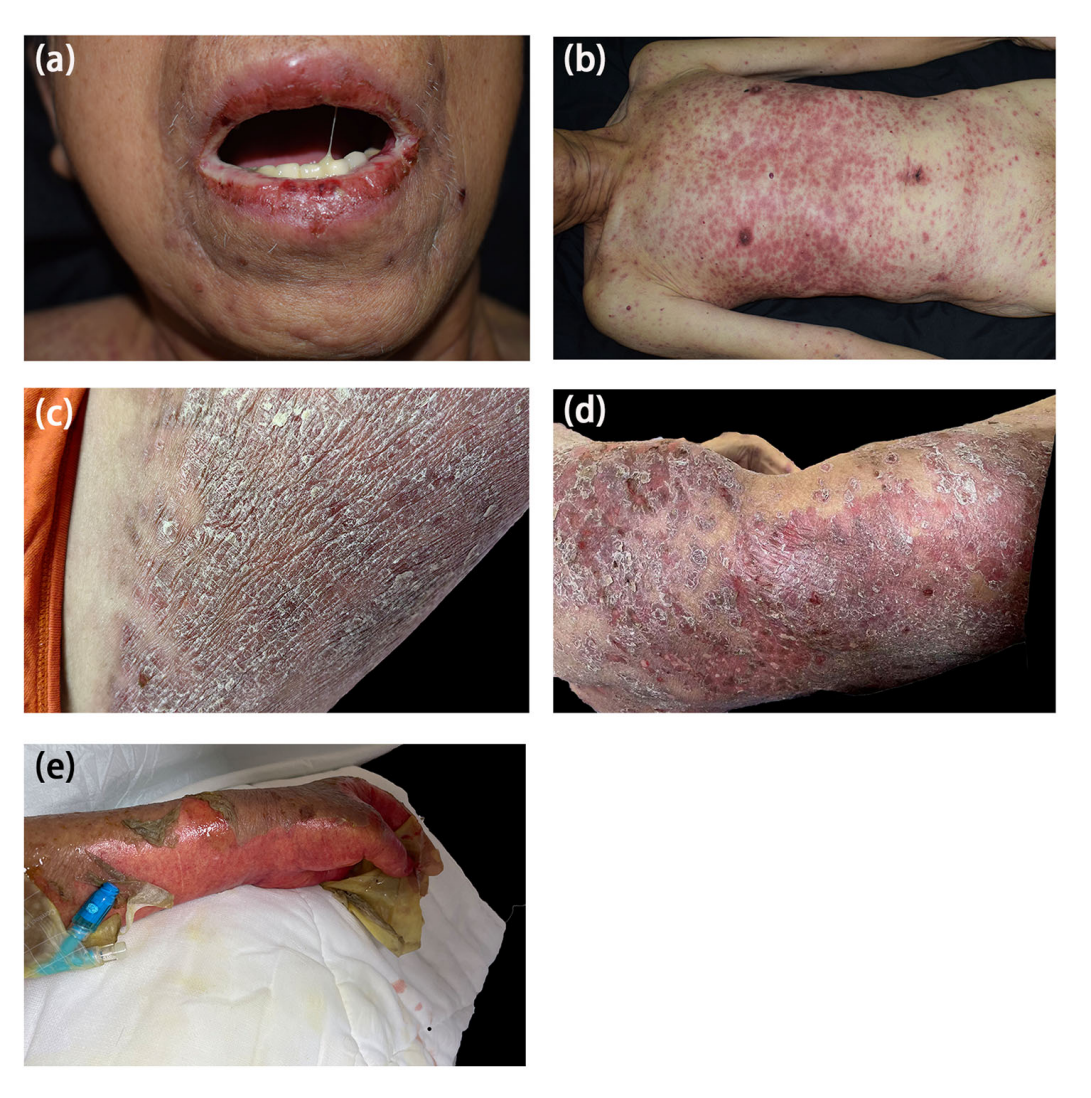
Clinical features. Case 1 **(A, B)** Buccal mucosa ulcers **(A)** and skin eruption on the trunk and limbs **(B)**. Case 2 **(C)** Skin eruptions on the limbs. Case 3 **(D)** Skin eruptions on the trunk. Case 4 **(E)** Multiple areas of denuded skin on the limbs were noted.

**Figure 2:**
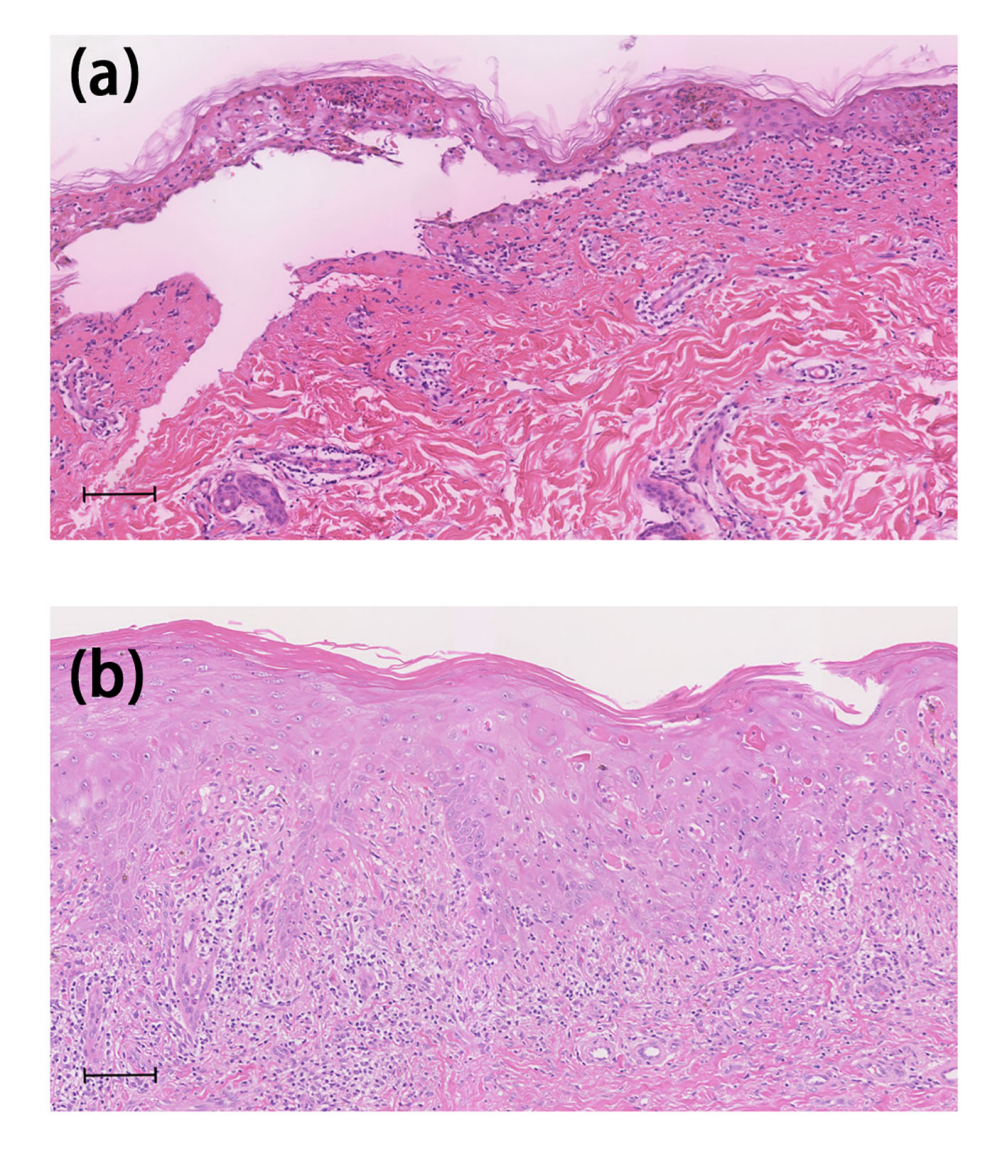
Histological features. Case 1 **(A)** Histological examination of the biopsy revealed interface dermatitis with whole-layer epidermal necrosis and dermal–epidermal separation and perivascular infiltration of lymphocytes in the upper dermis. Case 2 **(B)** Histopathological examination showed severe interface dermatitis with confluent apoptotic keratinocytes and perivascular lymphocyte and eosinophil infiltration was observed in the upper dermis. The scale bar indicates 100mm.

**Figure 3:**
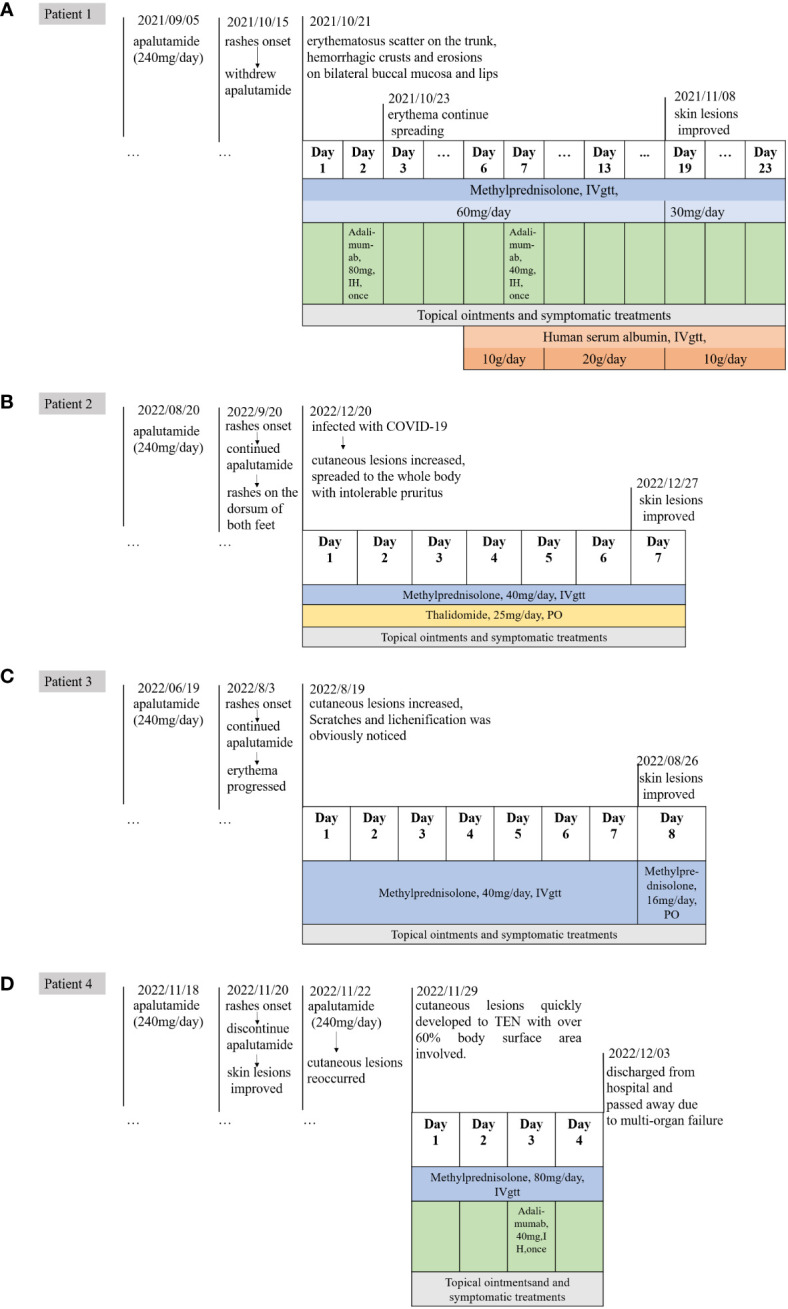
The timeline of medication history and treatments of Case1-4. **(A)** The timeline of case 1. **(B)** The timeline of case 2. **(C)** The timeline of case 3. **(D)** The timeline of case 4.

One 77-year-old male patient with PC developed crimson maculopapular rashes on his trunk and limbs with buccal mucosal ulcers and ran a low-grade fever approximately 40 days after the initiation of apalutamide (240mg/day). His previous medical history included diabetes, chronic hepatitis B virus infection, which were treated with sitagliptin phosphate/metformin hydrochloride, tenofovir disoproxil fumarate and traditional Chinese medicine. These drugs were prescribed for more than 3 years without changes. The Naranjo adverse drug reaction probability scale score for apalutamide was 6 whereas that for other medications was 0, which indicated that apalutamide was the most probable drug to be blamed. Although the patient immediately withdrew apalutamide, the diffuse erythema continued spreading. Physical examination revealed numerous erythematosus scattering on the trunk; besides, hemorrhagic crusts and erosions were noticed on bilateral buccal mucosa and lips (Body Surface Area (BSA) about 26%) (**Figures 1A, B**). Biopsy was performed indicating interface dermatitis with whole-layer epidermal necrosis and dermal-epidermal fissure along with superficial perivascular infiltration of lymphocytes (**Figure 2A**). A diagnosis of SJS was made, and we initiated intravenous methylprednisolone (60mg/day, tapered to 30mg/day after improvement of conditions) and human serum albumin, TNF-α inhibitor (Adalimumab, 80mg the first dose followed by 40mg one week later), as well as topical ointments and other symptomatic treatments, the skin lesions thereafter improved rapidly (**Figure 3A**).

### Case 2

One 69-year-old male patient who denied any allergies or previous medical history, developed florid papules and plaque on the dorsum of both feet for three months. The cutaneous lesions gradually increased, spreading to the whole body with intolerable pruritus (BSA, 34%) (**Figure 1C**). He started apalutamide (240mg/day) four months ago and he denied any other suspicious medication history even if the resident physician asked repeatedly. The Naranjo scale score for apalutamide was 6 which indicated that apalutamide was the probable cause. Of note, the rash worsened when he underwent COVID-19 infection. Histopathological findings were consistent with the diagnosis of drug eruptions including epidermal necrosis and perivascular infiltrated lymphocytes and eosinophils in the upper dermis (**Figure 2B**). Once he was hospitalized, a diagnosis of SJS was made and apalutamide was quit, thereafter, intravenous methylprednisolone (40mg/day) and oral thalidomide (25mg/day), as well as topical ointments were prescribed subsequently. Within two weeks, the skin lesions were better off and no relapse was heard (**Figure 3B**).

### Case 3

One 72-year-old male patient with PC developed maculopapular erythema on his whole-body excluding head, neck and mucosa for around 45 days after the start of oral apalutamide (240 mg/day). He refused to discontinue apalutamide, hence erythema progressed by degrees and pruritus intensified. Scratches and lichenification were obvious (BAS about 66%) (**Figure 1D**). He had no comorbidities and negated any other drug history or anaphylactic conditions. The Naranjo scale score for apalutamide was 6 which indicated that apalutamide was the probable cause of the drug eruption. SJS was diagnosed and systemic methylprednisolone (40mg/day) were given and the skin rashes were better off. However, the patient requests discharge hence systemic corticosteroids were altered to oral tablets. On regular visits, oral medrol was tapered gradually and luckily, he reported steady improvement (**Figure 3C**).

### Case 4

One 74-year-old male patient started apalutamide (240<no></no> mg/day) after the diagnosis of castration-resistant prostate cancer. His previous medical history included hypertension, diabetes, chronic kidney disease stage 5, renal anemia, chronic hepatitis B virus infection, hepatic insufficiency. No other newly-added drugs other than apalutamide were prescribed 3 months prior to such the condition. Shortly after the initiation of the drug (some 2 days, the patient cannot recall exactly), he experienced nausea and late-onset erythema on his trunk. He denied any adjustments of other daily drugs. Apalutamide was immediately discontinued and regular systemic treatments were prescribed subsequently and the rashes were on the mend. Unfortunately, owing to the rapid improvement of the lesions, the urologist advised him to recommence the apalutamide treatment for his prostate cancer. Thereafter his cutaneous lesions reoccurred and quickly developed to TEN with over 60% BSA involved. Moreover, necrosis of buccal and urethral mucosa was significant with exudation (**Figure 1E**). The Naranjo scale score for apalutamide was 9, indicating that apalutamide was the culprit. After admission, a diagnosis of TEN was made and the patient’s severity-of-illness score for TEN (SCORTEN) was 5, which indicated a quite poor prognosis. Though in-time and powerful medical care including large dose methylprednisolone (80mg/day) and 80mg adalimumab once was given, he passed away due to multi-organ failure within 3 days (**Figure 3D**).

## Discussion

According to global SPARTAN and TITAN studies, the incidence of skin rashes in the apalutamide group was as high as 23.8% and 27.1%, respectively (1). Although cutaneous lesions being considered a common adverse event in clinical trial studies, only a few reports describe the real-world features of apalutamide-induced drug eruptions. Herein, we reported a series of severe dAEs from China to emphasize the awareness of severe or even lethal prognosis when initiating apalutamide in clinical practices. Serious dAEs comprise of SJS, TEN, and drug rash with eosinophilia and systemic symptoms(DRESS). Although apalutamide has been reported to contribute to DRESS (21), the documented four patients did not have elevated eosinophils, fever or visceral damage such as abarrent liver enzymes.

**Figure 4:**
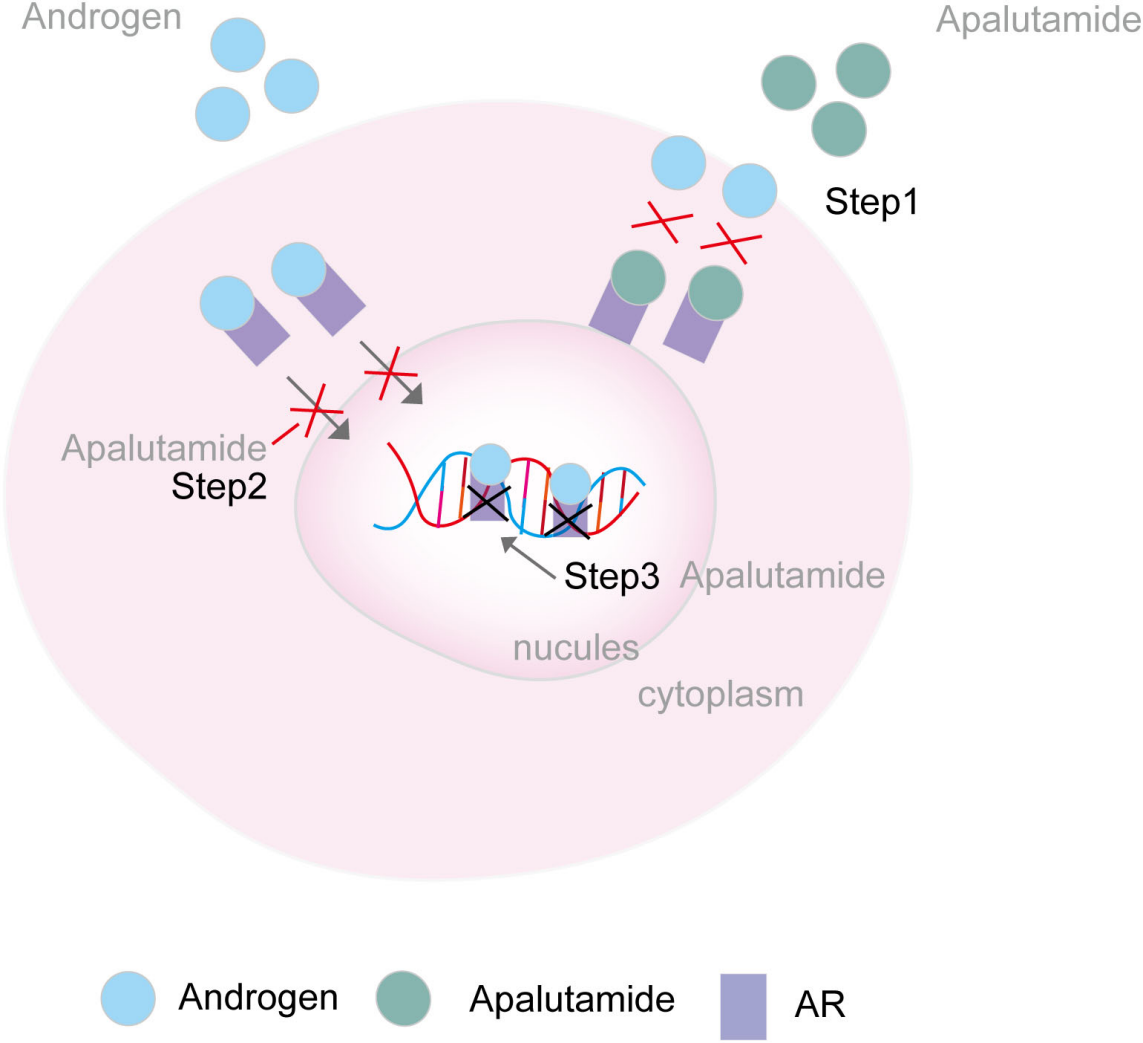
Mechanism of AR inhibitors. Step 1. Apalutamide binds directly to the AR ligand-binding domain and prevents AR activation; Step 2. Apalutamide inhibits the translocation of AR in the nucleus. Step 3. Apalutamide inhibits the transcription of target genes via preventing the AR and DNA incorporation AR, androgen receptor.

Apalutamide is a second-generation, selective inhibitor of the androgen receptor (AR) developed by Aragon Pharmaceuticals, Inc. It employs a tri-modal mechanism of action: binds directly to the AR ligand-binding domain (AR-LBD) to prevent AR activation, inhibiting the translocation of AR into the nucleus, and obstructing the transcription of target genes by preventing AR and DNA incorporation (**Figure 4**). This, in turn, induces tumor cell death (22). In contrast to apalutamide, other nonsteroidal androgen receptor inhibitors, such as enzalutamide, are not commonly associated with a high incidence of skin rash. Apalutamide’s chemical structure, when compared to enzalutamide, features a more reactive 2-cyanopyridine component that could more readily activate the immune system, leading to increased lymph node cellularity, T-cell, and B-cell counts. Data from Changhua Ji’s team supports the hypothesis that the 2-cyanopyridine moiety in apalutamide may react with cysteine in proteins, forming haptens that could trigger an immune response. This immune response, as indicated by apalutamide’s activity in the MDAM assay, may contribute to the increased potential for skin rash in patients compared to those on a placebo, as observed in the SPARTAN and TITAN clinical trials (23). Their hypothesis is consistent with Yoichiro Tohi et al., who suggested that the apalutamide-associated skin rash might not be attributed to allergic reactions but rather to a structure-specific, off-target pharmacological reaction (12). Additionally, Michie Katsut et al. found that low body weight is a risk factor for apalutamide-related cutaneous adverse events (24).

Compared to many other drugs, the incubation period of apalutamide is relatively longer with a reported median time of 82 days according to the SPARTAN trial and 80.5 days according to the TITAN trial (1). In this case series, the median time interval was around 40 days while the longest incubation period around 150 days. Long life-span and atypical cutaneous features may contribute to such phenomena. It is postulated that apalutamide has a propensity to bind to serum proteins, allowing it to circulate in the body. Clinical trial data have indicated detectable drug molecules in plasma even after a single dose of 240mg for up to 71 days (8). Therefore, with the expanded approval of apalutamide in China, dermatologists need to vigilantly assess cutaneous symptoms in patients undergoing apalutamide treatment, especially within the first 6 months of initial administration. Furthermore, the immediate discontinuation of apalutamide is crucial in managing drug-related adverse events (dAEs). Rechallenge, even at a lower dose, may be unsafe and potentially life-threatening. Similar to the patient mentioned in ‘Case 4,’ individuals who may be allergic to apalutamide should promptly consult with their urologist to establish a further medication plan based on a mutual understanding of such conditions.

Systemic corticosteroids, immunodepressant, intravenous immunoglobulin and plasmapheresis are of great benefit to dAEs. In the past few decades, plasmapheresis also performed in patients of dAEs, however, studies have pointed out that the use of plasma exchange to treat TEN does not improve the mortality, duration of the disease, or skin healing time (25). In patients with TEN, there is a notably high expression of TNF-α in both plasma and cutaneous blister fluids. Additionally, TNF-α is overexpressed in keratinocytes, potentially inducing keratinocyte apoptosis through the caspase cascade and Fas/Fas ligand interaction (25). These findings demonstrate the initiation of TNF-α inhibitors could shorten the re-epithelization time and thus promote skin healing significantly (14). For instance, studies by Hunger et al. and Wang et al. have demonstrated that TEN patients treated with TNF-α inhibitors experienced faster recovery compared to those treated with immunoglobulins or corticosteroids (25). Thus TNF-α inhibitor may be a promising treatment option in the management of apalutamide associated TEN.

## Conclusion

While the overall prognosis of severe drug-related adverse events (dAEs) may be discouraging, prompt withdrawal of the medication without rechallenge can make the difference in saving a life.

Special and adequate attention should be paid to apalutamide-attributed severe cutaneous adverse effects. The early and accurate diagnosis of apalutamide-related drug eruption is vital for the ultimate prognosis of the patient, and maintaining close contact with dermatologists is indispensable. Though overall prognosis of severe dAEs may be disappointing, prompt withdrawal of the medication and without rechallege may be life-saving.

## Data availability statement

The original contributions presented in the study are included in the article/supplementary material. Further inquiries can be directed to the corresponding author.

## Ethics statement

The studies involving humans were approved by Human Ethics Review Committee of the Second Affiliated Hospital to Zhejiang University. The studies were conducted in accordance with the local legislation and institutional requirements. The participants provided their written informed consent to participate in this study. Written informed consent was obtained from the individual(s) for the publication of any potentially identifiable images or data included in this article.

## Author contributions

QW: Writing – original draft, Writing – review & editing. HC: Writing – original draft. XZ: Writing – original draft. HW: Writing – original draft. ZT: Writing – review & editing.
